# Evaluating the well-being of ENT trainees in the UK: survey findings

**DOI:** 10.1017/S0022215125103848

**Published:** 2026-01

**Authors:** Vanessa Baxter, Tharsika Myuran, Winifred Eboh, Reza Majdzadeh, Frederick Green

**Affiliations:** 1 School of Health & Social Care, University of Essex, when research was conducted, now Institute for Health and Care Improvement, York St John University, Colchester, UK; 2 Association of Otolaryngologists in Training, UK; 3 School of Health & Social Care, University of Essex, UK

**Keywords:** otolaryngologists, psychological well-being, burnout, professional, inservice training

## Abstract

**Objective:**

The Association of Otolaryngologists in Training wanted to assess trainee well-being.

**Methods:**

A survey was developed that incorporated the Copenhagen Burnout Inventory, the short Warwick–Edinburgh Mental Wellbeing Scale and the Brief Resilience Scale plus questions on working conditions.

**Results:**

There were 190 responses and while most respondents had low or moderate levels of burnout, 15 per cent had high personal burnout and 13 per cent had high work-related burnout. The mean well-being score for respondents was lower than for the whole population mean. In addition, 39 per cent of respondents reported their mental well-being had been slightly affected in a negative way by their working environment and conditions in the last 6 months, and 26 per cent reported it being significantly affected negatively. Of these, 43 respondents reported an impact on patient safety.

**Conclusion:**

This first-ever survey of ENT trainees in the UK identified several areas of concern, including how the working environment and conditions affect trainee well-being and impact patient safety.

## Introduction

The Association of Otolaryngologists in Training in the UK represents all otolaryngology and head and neck surgery (ENT) trainees in the country. It is an independent organisation and is run by trainees, for trainees. The Association of Otolaryngologists in Training Forum has around 2000 members, including junior doctors of all grades, with at least 350 registrars in training to become consultants.

The Association of Otolaryngologists in Training undertook a survey of ENT trainees in 2022, assessing trainee well-being in their ENT posts and including sections on bullying and harassment. They commissioned the University of Essex to run the survey, analyse responses and report on its findings.

Doctors are known to be at higher risk of anxiety, depression, suicide and substance abuse when compared with the general population.^1^^–^^3^ A review on occupational diseases amongst UK surgeons^4^ found that surgeons in the UK are likely to be susceptible to stress, burnout and psychiatric morbidities, substance abuse, sharp injuries and musculoskeletal pain.

Factors contributing to this include a high-pressured work environment resulting in high levels of stress, long working hours, a heavy clinical and managerial workload, the perceived poor handling of clinical and professional issues by management, raised patients’ expectations and feeling under-resourced. All of these can have an adverse effect on both physical and mental well-being.^4^^,^^5^

A recent qualitative study also identified several other sources of work-related psychological distress experienced by foundation and junior doctors in the UK: toxic or unsupportive work cultures, including bullying and abuse, sexism and racism, and a culture of blaming and shaming individuals (often junior staff) for any medical errors; the lack of psychological or emotional support, particularly from other team members, consultants and supervisors; and the stigma associated with mental health issues, work-related burnout and stress, with the perceived need to appear invulnerable within medicine, where work-related stress and distress are normalised and taking time off for mental health is poorly tolerated.^6^

Negative impacts on well-being include ‘burnout’, a term coined by Freudenberger in 1974^7^ and which the World Health Organisation^8^ describes as:

‘A syndrome conceptualised as resulting from chronic workplace stress that has not been successfully managed. It is characterised by three dimensions: feelings of energy depletion or exhaustion; increased mental distance from one’s job, or feelings of negativism or cynicism related to one’s job; and reduced professional efficacy.’

In a 2018 survey of doctors and students, the British Medical Association^9^ found that 80 per cent of doctors who responded were at high and/or very high risk of burnout, with junior doctors being most at risk. It also found that 27 per cent of respondents reported being diagnosed with a mental health condition at some point in their life and 40 per cent reported they were currently suffering from a broad range of psychological and emotional conditions. Doctors who worked the longest weekly hours (51 or more hours per week) were most likely to say they were currently suffering, and 90 per cent said their current working, training or studying environment had contributed to their condition to either a significant or a partial extent.^9^ In May 2020, the British Medical Association found that 45 per cent of doctors were suffering from depression, anxiety, stress, burnout or other mental health conditions relating to, or made worse by, the coronavirus disease 2019 (Covid-19) crisis.^10^

These mental health and well-being issues can affect a doctor’s job performance and decision-making ability, which in turn could have a negative impact on patient safety.^11^ Doctors affected by burnout are more likely to take early retirement, which could result in a significant reduction in the number of doctors working within the National Health Service and could also impact on the training of future generations of doctors.^4^^,^^12^^,^^13^

Few studies on well-being have been carried out amongst ENT surgeons or trainees, either in the UK or elsewhere. A systematic review of published literature^14^ (primarily from the USA) was undertaken to characterise current trends of burnout and well-being among otolaryngology trainees and found that rates of burnout remain high among these trainees. The total hours worked per week and being female were associated with reduced well-being. Implementing protected non-clinical time and formal trainee mentorship programmes have been shown to decrease burnout and stress, and to increase well-being among trainees. In qualitative studies, trainees reported increased levels of distress and emotional hardening compared with attending otolaryngologists. In quantitative studies, burnout rates were high among ENT trainees, although they appear to be improving over time.

A recent literature review^15^ looking at the UK’s junior doctor workforce retention crisis identified that working conditions were a key determining factor, along with support and relationships plus learning and development, with lack of flexibility as an overarching theme. This leads to junior doctors not feeling valued, having a poor work–life balance, lacking autonomy and providing compromised patient care.

Several problematic working conditions were identified: (1) high workloads, which were exacerbated by rota gaps and excess administrative work; (2) long and antisocial working hours, often with longer hours as a result of staff shortages and an excessive workload, leading to a lack of regular routines and the completion of training records being done outside of working hours; (3) having insufficient time frequently during shifts to eat, drink and rest or having breaks disrupted; (4) rotas often being distributed at late notice or with last-minute changes so that it is difficult to plan life outside of work, with gaps in rotas being common and not managed appropriately, which results in junior doctors being pressured to work additional hours or above their grade and difficulties in obtaining annual or study leave at the desired time, including for important family events such a funeral or even their own wedding; and (5) poor facilities with insufficient space for engaging with their team, inconsistent provision of break rooms during and after shifts (some of which require payment), problematic information technology systems and WiFi, and a lack of canteens, parking and accommodation.

## Aim of the study

The Association of Otolaryngologists in Training wanted to assess trainee well-being in their ENT posts.

## Materials and methods

### Methodology

The Association of Otolaryngologists in Training designed an online survey that was reviewed by the University of Essex with suggestions for additions and minor amendments. The survey included questions on working conditions for ENT trainees, followed by several previously validated inventories evaluating well-being, burnout and resilience: the short Warwick–Edinburgh Mental Wellbeing Scale,^16^ the Copenhagen Burnout Inventory^17^ and the Brief Resilience Scale.^18^ Demographics, including UK region, age, level of training, gender, sexual orientation, reported disability, ethnicity and religion were also recorded.

### Recruitment

An online survey link was circulated to Association of Otolaryngologists in Training forum members via email and the participation of respondents was voluntary. At the end of the survey, respondents were given links to sources of support and information.

### Participants

The survey was live between October and December 2022, with 190 responses received, which gives a response rate of 54.3 per cent. This is similar to the average response rate of 53.3 ± 24.5 per cent (mean ± standard deviation (SD)) achieved by 1746 online surveys of healthcare professionals.^19^

Of these 190 survey respondents, 81 per cent were registrars, 44 per cent were male (*n* = 88) and 41 per cent were female (*n* = 82), while the remainder reported they were non-binary or preferred not to say. It was found that 86 per cent (*n* = 153) of respondents were straight heterosexual while 3 per cent (*n* = 6) were gay or lesbian and 3 per cent (*n* = 5) were bisexual (the remainder preferred not to say). Finally, 79 per cent of respondents (*n* = 147) were aged between 30 and 39 years.

Two thirds of respondents said that they did not work less than full-time, with 17 per cent of the female respondents saying that they worked less than full-time under category 1 (disability and/or ill-health and/or caring responsibilities) compared with 3 per cent of male respondents, and 30 per cent of female respondents saying they did not work less than full-time but would like to compared with 15 per cent of male respondents.

### Data analysis

Survey responses were analysed using Excel and SPSS.

The statistical analysis included both descriptive and analytical methods. The descriptive method utilised categories of well-being to demonstrate the respondents’ situation, including non-respondents. Numbers and percentages are reported for each category to emphasise the importance of well-being.

Descriptive statistics were used to analyse categorical variables. This utilised scores from the questionnaire and reports the mean, SD and standard error. Because the responses were categories and did not have a normal distribution, means between demographic groups were compared using the non-parametric Kruskal–Wallis for multiple group comparisons and Mann–Whitney for two groups, with significance levels considered at *p* equal to 0.05. Because of the number of statistical tests for pairwise comparisons, significance values were adjusted using the Bonferroni method.

### Limitations

An online survey has several limitations. Firstly, the response rate cannot be accurately determined because it is impossible to know precisely how many trainees viewed the email invitation. A lower response rate decreases the generalisability of the findings. Although the total population of ENT trainees is unknown, 190 responses out of an estimated 350 Association of Otolaryngologists in Training forum members gives an estimated 54.3 per cent response rate.

Secondly, a sampling bias amongst responders is possible because they are self-selecting as participation was voluntary. This may further affect the generalisability of findings. This is an interesting point because many surveys of healthcare professionals (e.g. General Medical Council (GMC), Intercollegiate Surgical Curriculum Programme (ISCP)) are mandatory.

### Ethics

Ethical approval was granted by the University of Essex’s Research Ethics Committee 2 (Reference: ETH2223-0035). Written, informed consent was obtained from all individual participants included in the study.

## Results and analysis

### Well-being

The Warwick–Edinburgh Mental Wellbeing Scale was developed to enable the monitoring of mental well-being in the general population and the evaluation of projects, programmes and policies that aim to improve mental well-being. The short version uses 7 of the Warwick–Edinburgh Mental Wellbeing Scale’s 14 statements about thoughts and feelings, which relate more to functioning than feelings and so offer a slightly different perspective on mental well-being. Scores range from 7 to 35 and higher scores indicate higher positive mental well-being.^16^

Using the Health Survey for England 2010–2013 (*n* = 27 169 adults aged 16 years and over, nationally representative of the population), norms were estimated and the mean score was 23.5 (23.7 for men and 23.2 for women).^20^ A survey of nurse, midwives and allied health professionals in May and July 2020, with 1410 respondents, reported well-being scores of 21.31.^21^

The mean well-being score for respondents to this survey was 22.8, which is lower than the mean score for the whole population (23.5) but higher than the mean scores for health professionals in summer 2020. There were no statistically significant differences in the scores by category of respondent.

Scores can be divided into high, average and low mental well-being using cut-off points at plus or minus one SD. This approach puts approximately 15 per cent of the participants into high well-being and 15 per cent into low well-being categories. Using this approach, UK population samples put the top 15 per cent of scores ranging from 27.5 to 35.0 and the bottom 15 per cent from 7.0 to 19.5.

While 15 per cent of the scores for respondents to this survey were in the range of 27.5 to 35.0 (in line with the population norm), 18 per cent were in the range of 7.0 to 19.5, which is 3 percentage points higher than the population norm.

It was found that 39 per cent of respondents (*n* = 59) reported their mental well-being had been slightly affected in a negative way by their working conditions, whilst 26 per cent (*n* = 40) said their mental well-being had been significantly affected in a negative way. In addition, 21 per cent (*n* = 32) of respondents said their mental well-being had not been affected, while just 6 per cent (*n* = 8) said it had been affected positively.

‘I have prioritised patient safety persistently, which means my own well-being has been neglected much of the time.’ (Female respondent)

‘I have found myself being more intolerant, and my concentration has been affected.’ (Respondent who did not report their gender)

Of these respondents, 43 reported that the detriment to mental well-being affected patient safety. Reasons for this included the impact of not having enough sleep and/or feeling tired, making simple and/or careless errors, and reduced efficiency and clarity of thought.

‘It affected my functioning at work such that I made simplistic errors I would otherwise not have, which would have potentially resulted in actual patient harm if I had not corrected them. I also had to take time off work, which affected clinical staffing in our department and hence potentially patient safety.’ (Female respondent)

‘Less enthusiasm at work, poor sleep which impacts on concentration and decision making.’ (Female respondent)

Overall, 30 per cent of respondents (*n* = 45) said they regretted their decision to become a doctor and 44 per cent (*n* = 65) said they have thought about giving up medicine for another career.

### Supporting well-being at work

The survey found that 61 per cent of respondents (*n* = 92) agreed that their workplace supports their well-being at work and 68 per cent (*n* = 104) agreed that they know where to get support if their mental well-being is affected. However, 11 per cent (*n* = 17) and 4 per cent (*n* = 7), respectively, strongly disagreed with these two statements.

There were 56 suggestions made to improve respondents’ well-being, with the main ones relating to: staffing levels and/or working conditions, being valued as an employee (or not being valued) and/or better understanding or support from consultants and managers, pay and finances, including their administration and the reimbursement of travel and relocation costs, and more or better support for training.

Sixteen suggestions related to being valued as an employee (or not being valued) and/or better understanding or support from consultant and managers.

‘A complete overhaul of NHS management so trainees were not treated like inanimate resources to fulfil numerical service provision.’ (Male respondent)

‘Trainers who care about you as an individual and who care about your training. The effort one puts in as a trainee is not reflected in the training one receives.’ (Male respondent)

‘Being treated like a human by admin staff (local and regional). The way that some admin staff treat junior doctors is horrific (rude, unhelpful, misogynistic) and there is no accountability. The way the Dean is allowed to reject mileage and there is no accountability for the possibility it may be an unfair decision. There has to be better systems in place if you are being mistreated.’ (Female respondent)

Fifteen comments related to pay and finances, including their administration and the reimbursement of travel and relocation costs.

‘Despite being a senior registrar, earning significantly less than my friend who is a train driver, who works less hours than me.’ (Male respondent)

‘Higher pay that recognises my skills, qualifications and output. Having management that doesn’t mess up simple things like our salary and pay. Being treated better as a doctor, shorter commute, better relocation expenses.’ (Male respondent)

‘Proper pay. Not having to fight with HR every time I have a change of hospital or my circumstances. It’s like the system is trying to underpay and overtax me and see if I notice it.’ (Male respondent)

Ten respondents wanted more or better support for training.

‘Have study leave and compulsory exam leave approved without having to beg and rearrange the whole department’s rota.’ (Female respondent)

Six suggestions related to work–life balance and having time for hobbies or family life, while three respondents wanted a shorter commute.

‘The main thing that negatively impacts my well-being is long commutes due to centres in the deanery being very spread out. It is impractical to move house every year. Long commutes in the car make the working day long and tiring. It means I can’t spend time with family and I don’t have energy to work on projects (QIPs, papers etc) at the end of the working day.’ (Female respondent)

Five suggestions made were to improve the working environment, including better food in staff canteens.

‘A dedicated room for registrars to work that is quiet, clean and not messy. Even just the addition of a plant would help… An on-call room which is pleasant and not freezing in the winter!’ (Female respondent)

### Burnout

The Copenhagen Burnout Inventory was developed with a framework that characterises the core of burnout as fatigue and exhaustion, which are attributed to specific domains in a person’s life (personal, work-related and client-related). It is a 19-item survey with positively and negatively framed items that covers 3 areas: personal (degree of physical and psychological fatigue and exhaustion), work-related (degree of physical and psychological fatigue and exhaustion related to work) and client-related (or a similar term such as patient, student, etc.) burnout.^17^ The mean scores for the 3 domains are classified as none and/or low (less than 50), moderate (50–74), high (75–99) and severe (100).^22^

An online survey of neurosurgical trainees in the UK and Ireland, with 75 respondents, had a median Copenhagen Burnout Inventory score of 38.85. Participants showed a higher degree of personal and workplace burnout (median Copenhagen Burnout Inventory scores of 47.02 and 49.14, respectively) compared with patient-related burnout (median Copenhagen Burnout Inventory score of 18.67).^23^ A survey of staff working in an acute paediatric hospital setting in Ireland during the Covid-19 pandemic, with 133 respondents, reported a mean score in the 3 domains of 56.9 for personal burnout, 55.6 for work-related burnout and 28.1 for patient-related burnout.^24^ A survey of nurses, midwives and allied health professionals in summer 2020, with 1410 respondents, reported a mean score of 58.82 for personal burnout, 54.67 for work-related burnout and 25.02 for patient-related burnout.^21^

Mean scores in this survey were 54.88 for personal burnout, 51.14 for work-related burnout and 25.58 for patient-related burnout. These are all higher than the scores (47.02, 49.14 and 18.67, respectively) measured for neurosurgical trainees in 2021 but are lower than the scores in summer 2020 for nurses, midwives and allied health professionals (58.82, 54.67 and 25.02, respectively).

Respondents who reported having a specific learning disability had a higher score for personal burnout (66.21 compared with 52.88, *p* = 0.025) and work-related burnout (62.66 compared with 49.29) (*p* = 0.015) than those who said they did not. Respondents aged 30–39 years had higher scores for patient-related burnout than those aged 20–29 years (27.32 compared with 21.25, *p* = 0.027).

While the majority of respondents had low or moderate levels of burnout on all 3 domains, 15 per cent (*n* = 32) had a high level of personal burnout, with 3 per cent (*n* = 4) having a severe level of burnout, and 13 per cent (*n* = 20) had a high level of work-related burnout. Just 1 per cent (*n* = 1) had a high level of patient-related burnout.

### Resilience

The Brief Resilience Scale was created to assess the perceived ability to bounce back or recover from stress. The scale assesses a unitary construct of resilience, including both positively and negatively worded items. The possible score range on the Brief Resilience Scale is from 1 (low resilience) to 5 (high resilience).^18^ In a study with 844 participants, a mix of healthy people and people suffering from diseases, Smith and colleagues found a mean score of 3.70.^25^ A study of paediatric and neonatal intensive care unit staff with 58 respondents (32 nurses, 22 doctors and 4 other healthcare professionals) reported a mean Brief Resilience Scale score of 3.58.^26^

The mean score on resilience for respondents to this survey was 3.41, which is lower than the 3.58 mean from the Dalia *et al*. study.^26^

Female respondents had lower mean resilience scores than male respondents (3.13 compared with 3.69, *p* = 002.), while respondents saying they had a specific learning disability had lower resilience scores than those who said they did not (2.85 compared with 3.51, *p* = 0.002). The Brief Resilience Scale was used to assess the self-perceived ability to bounce back or recover from stress. The mean score for respondents was 3.4, which is lower than the 3.7 mean from a national study of patients.^24^ Furthermore, 8 per cent of respondents (*n* = 12) showed a high level of resilience and 67 per cent (*n* = 98) showed a moderate level of resilience, but 25 per cent (*n* = 36) showed a low level of resilience.

Female respondents had lower mean resilience scores than males, while those who reported a specific learning disability had lower resilience scores than those who did not.

It was found that 8 per cent of respondents (*n* = 12) were measured as having a high level of resilience and 67 per cent (*n* = 98) had a moderate level of resilience. However, 25 per cent of respondents (*n* = 36) had a low level of resilience.

### Working conditions

A quarter of respondents (*n* = 36) reported that their work schedule reflected the hours they actually work. Another 36 per cent (*n* = 51) worked up to 5 hours extra per week, 25 per cent (*n* = 36) worked 5–10 hours extra and 8 per cent (*n* = 11) worked over 10 hours extra per week.

It was found that 87 per cent of respondents (*n* = 160) worked resident on calls and 39 per cent of respondents (*n* = 75) reported that when working non-resident on call they lived within on-call distance from their hospital, while 39 per cent (*n* = 76) did not.

Just over a quarter of respondents (*n* = 46) reported that if working non-resident on call, they were not given time off the next day following less than 6 hours of continuous rest during the on-call shift. Just 15 per cent of respondents (*n* = 27) reported being always supported in having time off the next day and 21 per cent (*n* = 37) said they were usually supported to do so.

In addition, 64 per cent of respondents (*n* = 110) reported that a dedicated on-call room was available where they worked, although it was not pre-bookable for a proportion, but 25 per cent of respondents (*n* = 43) said that there was no such room. Just over a third of respondents (*n* = 57) reported that, on average in the last month, an on-call room was always available, with another 8 per cent (*n* = 14) saying one was frequently available, but 19 per cent (*n* = 32) reported that an on-call room was never available. When no on-call room was available, 24 per cent of these respondents (*n* = 26) paid for a hotel (some of whom were then reimbursed), 24 per cent (*n* = 23) slept in their office and 15 per cent (*n* = 16) went home.

‘Some hospitals provide on-call rooms, not all. Some are not on site at the hospital.’ (Male respondent)

‘If we get chance to sleep when working overnight it is on a mattress on the floor of our tiny office.’ (Female respondent)

The survey found that 30 per cent of respondents (*n* = 45) reported feeling completely safe, secure and comfortable using the on-call accommodation and 29 per cent (*n* = 44) mostly felt safe. However, 29 per cent (*n* = 43) reported that they did not feel safe, secure and comfortable using the on-call accommodation. Suggested improvements to on-call accommodation included locks on doors, or better locks, better comfort or a more comfortable bed and/or warm bedding, ensuite or non-communal bathrooms, better lighting and/or security support, cleaner accommodation and accommodation being nearer to the hospital or wards.

‘On several occasions, someone has entered the room when I was asleep in there. Estates have now put a chain on the door but I always worry that the code is known by so many people and someone might already be in there when I go in late at night, as has happened before despite me booking the room.’ (Female respondent)

‘It is however just miserable. Too hot. Poor kitchen facilities. Nowhere to sit comfortably to sit and eat food (there used to be a living room but this was converted into another executive committee room and taken away from doctors).’ (Male respondent)

A total of 55 per cent of respondents (*n* = 77) reported that there was no free parking when travelling to a different site for emergencies whilst 17 per cent (*n* = 25) reported there was always free parking and 17 per cent (*n* = 25) reported that there was free parking most of the time. When travelling to another site for emergencies, 87 per cent of respondents (*n* = 60) had paid for parking out of their own pocket. Three quarters of respondents reported difficulties with parking at work, with 63 per cent (*n* = 93) reporting insufficient parking for staff, 46 per cent (*n* = 68) paying a premium fee for parking, 42 per cent (*n* = 62) not being given a parking permit and 26 per cent (*n* = 35) reporting unsafe places to park.

Just 15 per cent of respondents (*n* = 22) felt that relocation expenses were sufficient, with 47 per cent (*n* = 71) reporting them as insufficient (the question was not applicable for 34 per cent, *n* = 51).

Furthermore, 90 per cent of respondents (*n* = 150) sought training opportunities on their days off to meet training requirements, with 11 per cent (*n* = 19) doing this every week and 19 per cent (*n* = 31) doing this once a month. Overall, 37 per cent of respondents (*n* = 40) said that they would not have met their training requirements without seeking training opportunities on their days off.

### Reporting problems with working conditions

When asked to whom respondents would feel confident in reporting problems with working hours or conditions, the main replies were their educational or clinical supervisor followed by trainee reps and via the GMC survey.

The survey found that 23 per cent of respondents (*n* = 36) said that they would not feel confident in reporting problems to anyone. The main reason for this was the fear of repercussions or being seen as a difficult trainee and/or not able to cope, ‘if you report you become a black sheep’. Other significant reasons were that no action would be or had been taken and the perception that it is expected as part of the job.

‘Reporting is never anonymous and you will be blamed forever, maybe seniors will even make your life difficult. I may report to BMA but to take action means to ID yourself.’ (Female respondent)

‘The culture of bullying and victimisation in our deanery is frightening. I wouldn’t dare report anything or speak out. Even just existing leads to horrific bullying by consultants well known for this behaviour who have never been held accountable.’ (Female respondent)

‘Backfired from previous experience. I learnt to be comfortable with being bullied on regular basis.’ (Female respondent)

‘Nobody cares about doctors working hours or working conditions including senior doctors. Seen as difficult or lazy if you report problems about this. Senior figures turn a blind eye.’ (Female respondent)

‘It’s expected for doctors to work extra hours without getting compensated and without being able to open their mouths to talk about it. We’re expected to work extra hours like slaves with no compensation.’ (Male respondent)

## Discussion

Research commissioned by the British Medical Association in 2020,^10^ following on from its 2018 survey of doctors’ and medical students’ mental health, identified five potential groups of risk factors for poor well-being that could, in some cases, lead to worsening mental health: (1) systemic factors identified included issues arising from poor processes and systems such as understaffing and rota gaps, a lack of flexibility and/or poor work–life balance, pressures to discharge patients from hospital and greater regulatory fears; (2) endemic factors were where issues such as learning to cope effectively with clinical risk and dealing with traumatic events or unexpected outcomes were a necessary reality of a medical role; (3) interpersonal factors derived from doctors’ relationships with their peers and included issues related to the stigma around mental health, the hierarchy, bullying, an erosion of peer support networks and the perception that doctors tend naturally to have type-A personalities (i.e. being perfectionists, having a fear of weakness or being seen to fail); (4) environmental factors are based on practical issues and are often linked to the workplace environment such as a lack of basic workplace amenities, an absence of breaks and the impact on junior doctors of training rotations; and (5) socio-cultural factors were identified from wider contextual factors outside of the medical profession, including an increase in patient self-diagnosis and patients’ expectations alongside doctors feeling increasingly undervalued by the public.

The report recommended that a long-term strategy to protect staff health and well-being should consider 10 points, which include a number that are pertinent to the findings from this survey. The first is that supporting the mental health and physical health of doctors and staff must be a top priority, and employers must take preventative action to protect staff from developing poor well-being and health in the first place. Another recommendation is to develop well-being strategies and create healthy workplaces that encourage regular breaks, provide access food and rest facilities 24 hours a day and 7 days a week, and support staff travel to work (e.g. with free car parking).

The monitoring of health and well-being issues is also needed to ensure that interventions are effective. Support must be inclusive, accessible and meet users’ needs, taking into consideration the diversity of staff and their differing experiences of mental health. This needs to include groups such as doctors with a disability and international medical graduates (who are new to medical practice in the UK), who face additional barriers to accessing support. Another recommendation is for the active encouragement of peer support and mentoring for doctors, which could include buddying up experienced and inexperienced workers, and setting up Schwartz rounds or Balint groups.

Sleep deprivation, a result of poor in-hospital on-call resting facilities, adversely affects well-being and mental function.^27^ This affects patient safety and there is a need to develop a system that enables junior staff to seek help for their mental health and well-being.

One potential limitation of this study was the response rate of 54.3 per cent, and gathering more responses would enable a deeper understanding of the challenges faced by trainees, particularly for those reporting sexual assaults, and the associated impact on their mental health and well-being. One recommendation from this paper’s authors is to run the survey again while publicising it more widely to generate a higher response rate, alongside a qualitative study to follow up and explore some of the issues in more detail. Replicating the study within other surgical specialities, because trainees are a vulnerable group, should also be explored.

- Doctors and surgeons are known to be at higher risk of anxiety, stress, burnout, depression, suicide and substance abuse when compared with the general population, and these mental health and well-being issues can affect job performance and decision-making ability, which in turn could have a negative impact on patient safety
- Few studies on well-being have been carried out amongst ENT surgeons or trainees, either in the UK or elsewhere
- The findings from this first-ever survey of ENT trainees identified a number of areas of concern
- Most respondents had low or moderate levels of burnout, but 15 per cent had high personal burnout and 13 per cent had high work-related burnout
- The mean well-being score for respondents was lower than for the whole population mean
- In the survey, 39 per cent of respondents reported their mental well-being had been slightly affected in a negative way by their working environment and conditions in the last 6 months, and 26 per cent reported it being significantly affected in a negative way, with 43 respondents reporting an impact on patient safety

## Conclusions

This first-ever survey of ENT trainees in the UK identified a number of areas of concern. While most respondents had low or moderate levels of burnout, 15 per cent had high personal burnout and 13 per cent had high work-related burnout. The mean well-being score for respondents was lower than for the whole population mean. Overall, 39 per cent of respondents reported their mental well-being had been slightly affected in a negative way by their working environment and conditions in the last 6 months, and 26 per cent reported it being significantly affected in a negative way. Of these, 43 respondents reported an impact on patient safety, citing examples.
